# A Dynamic Prediction Neural Network Model of Cross-Border e-Commerce Sales for Virtual Community Knowledge Sharing

**DOI:** 10.1155/2022/2529372

**Published:** 2022-10-10

**Authors:** Li Tian, Xiumei Wang

**Affiliations:** Business School, Nanfang College Guangzhou, Guangzhou 510970, China

## Abstract

In this paper, a neural network algorithm is used to conduct in-depth research and analysis on the sales dynamics prediction of virtual community knowledge sharing in cross-border e-commerce. Both the expected returns and the social network structure are analyzed, and both have positive effects on knowledge sharing in the actual development process, but the degree of them also possesses certain variability. A model of the factors influencing the quality of knowledge shared by users is constructed to explore the relationship between the dimensions in social capital and how they affect the community users' perceptions of knowledge quality. Exploring the strong influencing factors of product repurchase rate has key implications for promoting product sales and sales forecasting. The scale of this paper has undergone several minor revisions, and the content validity is very good. Criterion validity is generally reflected by Person correlation coefficient, and construct validity includes exploratory factor analysis and confirmatory factor analysis. First, the values were clustered, and the optimal variables were selected using stepwise regression and fitted with Poisson regression models to explore the relationship between the repurchase rate of different products and the factors that strongly influence the repurchase rate in the case. To predict the sales of goods, the advantages of the BP neural network, LSTM neural network, and Verhulst gray model are combined: BP neural network can predict sales by combining the current data corresponding to independent variables, LSTM neural network can explore the influence of historical data on sales, and Verhulst model can predict sales based on the growth trend of variables. A BP-LSTM-Verhulst nested neural network based on the AP algorithm is constructed to predict sales volume, and the accuracy of this method is proved by example. Finally, it is found that the proposed sales prediction method has higher accuracy than exponential regression and shallow neural networks. The deep learning prediction method combining unstructured data such as images proposed in the paper not only provides a more accurate sales prediction method for short life cycle products in e-commerce but also provides an effective deep learning method for management practices. The KMO value obtained after the test is less than 0.5, which is not suitable for factor analysis. Using SPSS22.0 to expand the KMO value and Bartlett's sphericity test, the KMO value is significantly greater than the minimum requirement of 0.5, the KMO value of self-efficacy is 0.826, and the KMO value of expected return is 0.870.

## 1. Introduction

In today's world, knowledge is gradually becoming an important force for enterprises to improve the core competitiveness of talents and continuously gain competitive advantages, and it plays an irreplaceable role in the sustainable and healthy development of organizations [1]. In recent years, knowledge management has gradually been paid attention to and studied by scholars in the field of management and has gradually become the focus of research. Knowledge has the characteristic of shared growth, and knowledge sharing refers to the stage in which knowledge providers in an organization summarize their knowledge and then transform it by using corresponding tools and channels, and finally knowledge gainers internalize and absorb it, which is a process of knowledge learning itself. During the development of an organization, knowledge sharing can effectively enhance the innovation ability of the organization and thus strengthen the core competitiveness of the organization and individuals. Therefore, a new model structure of the recurrent neural network is introduced, which gives the neural network the function of memory and can obtain the influence of historical information on the current prediction. Contemporary Internet technology has shown vigorous development, and e-commerce is its representative product, which is a brand-new development direction of network technology application. Merchandise mix and sales forecasting have always been the core of e-commerce and an important prerequisite for the healthy development of enterprises and have a positive impact on market orientation and inventory control [2]. In recent years, the competition among e-commerce enterprises has become increasingly fierce. To occupy a favorable position in the market, it is necessary to make accurate predictions and judgments on the competitiveness of goods, and reasonable predictions will bring great economic benefits and maintain the stable development of the market, among which the research of effective methods of merchandise mix and sales forecasting is an urgent problem for current e-commerce enterprises.

With the enhancement of computer functions and the emergence of more machine learning techniques and statistical forecasting methods, the field of demand forecasting is booming. People are consuming increasingly, and the level of material consumption is gradually increasing, leading to an increase in the frequency of consumption and prompting a gradual shortening of product life cycles [3]. For example, the split seasons of clothing in fast fashion products have evolved from the traditional four seasons to multiple seasons, and the average shelf life of fashion apparel, satchels, shoes, and hats produced by fast fashion product companies is often only a few weeks. Short life cycle products cannot be active in the market for a long time and are easily replaced by homogeneous competitive products, showing the characteristics of a short sales life cycle and being easy to depreciate. For some fast fashion products, such as shoes and hats and apparel fashion, because the product life cycle is relatively short, its demand forecast is more challenging. Forecasting methods look for trends or seasonal patterns in historical sales data and sometimes correlate them with events from other sources. However, when a new product is introduced for launch, there is little historical data available other than known information such as attributes [4]. This study discusses fast fashion products with short life cycles and focuses on retail-oriented companies that forecast the demand as well as sales of fast fashion products. The most important product characteristics of fast fashion products are fast feedback on showroom design, short product life cycle, and higher frequency of updating product categories and types. It is mainly for products that are mainly sold in offline stores. Fast fashion products as an important part of the manufacturing and retail industry not only represent the soft power of the country but also bring important support and influence to the stability and development of the national economy.

In past studies, there is no lack of research on product sales forecasting methods, and the goal of scholars mainly focuses on how to use time series methods to discover the trend of sales data from historical sales data, to predict the trend of future sales. However, time series methods have high requirements for the smoothness of the data. Each consumer in the market is supposed to be random and disorderly; however, time series methods will fail when some factors appear to make the whole or part of consumers appear to have a certain purchasing tendency; for example, promotional tools and other factors that affect users' purchasing decisions appear. Each user's purchase decision behavior ultimately constitutes the overall sales volume, but in the traditional sales forecasting research methods, due to the difficulty of obtaining individual purchase decision influencing factors, therefore, individual purchase decision influencing factors are rarely used to study sales forecasting. Currently, in the Internet environment, users' browsing and purchasing processes are recorded in full, and companies can search for factors influencing users' purchasing decisions based on their behavioral records and use the factors influencing users' purchasing decisions to make product sales forecasts.

## 2. Related Works

For quantitative methods, companies tend to use time series methods for forecasting. Time series methods are operable for forecasting sales, but they lack consideration of factors that affect product sales. Many scholars have introduced factors affecting users' purchasing behavior as influencing factors of sales and have achieved better results [5]. In the study, Thomasson predicted the sales of apparel companies for one-quarter, but it is difficult to guide the ordering behavior of companies in the medium and long term compared to the short life cycle of apparel. Mumu and other scholars proposed a crown model for the short-term sales problem of online agricultural products, combined with deep learning algorithms, and used simulated data to analyze and obtain better results [6]. The study uses the self-coding function of neural networks to encode and reduce the dimensionality of category variables such as shipping place, which effectively reduces the dimensionality of high-dimensional data and uncovers the correlation of different categories [7]. The stacked autoencoding neural network can learn in real time and improve the shortcomings of random initialization of the neural network. The study collected egg sales data from Taobao and considered the influence of information such as discounts, packages, and other promotional tools on users' purchasing behavior. The study shows that the accuracy of phased sales prediction is higher than that of direct prediction using neural networks [8].

Cao et al. designed a hybrid learning algorithm based on multi-intelligent body theory and social networks to analyze the knowledge sharing of virtual community users with the help of NetLogo 5.0 [9]. Pananond et al. propose the demand for personalized services in information-sharing virtual spaces and the demand knowledge base construction based on multiagent technology and discuss the characteristics and implementation scheme of agent technology [10]. On the Internet, there is often a large amount of information, which makes it more difficult for network users to search for the information they need and consumes the energy of community users to search for information, and the “relationship” chain communication mode of SNS provides a new way for people to obtain information. With its unique advantages of communication and knowledge sharing among community members, social network services have attracted extensive attention and research from a wide range of scholars [11]. He et al. put forward the concept of knowledge sharing itself, which pointed out that, among knowledge owners and demanders, information communication is used to help them complete the learning of knowledge, and the learning content mainly covers the knowledge and experience possessed during the learning period [12]. In the form of knowledge sharing, the process clarifies the importance of the channel itself, while pointing out that the use of certain tools can enhance the effectiveness of knowledge sharing.

When there is no consumer shopping, the vending machine must be connected to the cloud platform so that the cloud platform can read the vending machine's operational status data at any time. When consumers are shopping, it is important to ensure that the vending machine is connected to the cloud platform stably, as a failure of the connection can directly result in a failed order transaction. In addition, the order data transfer must be accurate, so a data verification service must be completed. From single models such as time series and artificial intelligence prediction methods to combined models, the empirical analysis proves that combined models can combine the advantages of single models and show high accuracy, and there is some inspiration for the method used in this paper. The predecessors have provided us with many valuable research methods, but the complexity of commodity forecasting itself predestines that there is more information value waiting for us to explore.

## 3. Analysis of the Factors Influencing Knowledge Sharing in Virtual Communities

The benefit-oriented rapid relationship includes three dimensions of mutual understanding, mutual benefit, and relationship harmony, among which, mutual benefit means that both parties feel that they will get positive benefits from establishing the relationship and can satisfy their respective interests, and this rapid relationship will prompt both parties to engage in continuous interactive behaviors. Relational commitment, interaction, and reciprocity play an important role in knowledge exchange, and long-term sustained behaviors are generated in relational virtual communities because they receive personal benefits during community participation and different experiential components influence sustained usage behaviors through perceived personal benefits. Knowledge sharing behaviors of users in virtual communities can improve their image and rank, as well as receiving favorable comments from other users, and psychological satisfaction and pleasure, and they are motivated to maintain long-term relationships to continuously obtain such benefits [13]. When the prediction generates a residual value, it starts to learn the residual value. There may be weak and long-term memory between the residual values. The corresponding accumulated residual series may not be stationary. Users sense that they will gain benefits from building relationships, and when the perceived benefits are strong, they are willing to maintain long-lasting relationships, which is an important motivation for sustained behavior. The relationships among users in academic virtual communities meet the definition of fast relationships and can explain users' willingness to sustain knowledge sharing.

**Figure 1:**
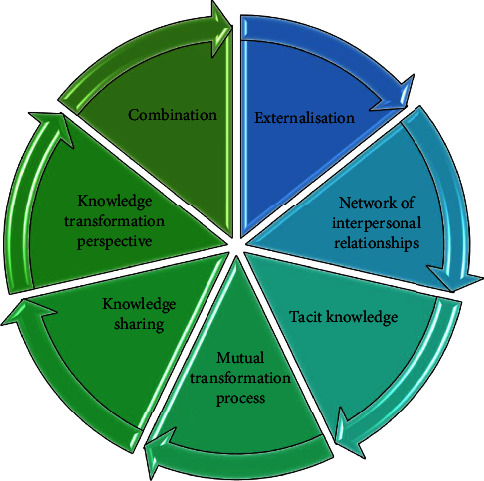
Knowledge sharing model of virtual community.

About the content of the knowledge transformation perspective, some studies point out that knowledge sharing can occur not only in individuals and organizations but also corresponding to the mutual transformation process in both explicit and tacit knowledge, and the key to the analysis of this transformation process, which is also called the SECI model, is the internalization process. Tacit knowledge is highly personal and therefore cannot be easily grasped or expressed by others. Therefore, the externalization of the knowledge owner's knowledge behavior plays a key role in the process of knowledge sharing. The virtual community social network realizes the sharing of information exchange across regions. The network of interpersonal relationships is a real opportunity to facilitate or limit knowledge sharing behavior, which makes it possible for individuals to shift from no relationships to weak or even strong relationships [14]. Most existing studies are based on a social network theory perspective, and they argue that network size, for example, affects knowledge sharing behavior to some extent. Knowledge sharing behaviors start to arise when knowledge owners within an organization perceive that the organization has a high level of organizational trust; on the contrary, when there is a trust crisis within the organization, the quantity and quality level of knowledge sharing becomes seriously problematic. Therefore, the social network structure and organizational trust are the main choices used in this study in terms of opportunity elements, as shown in Figure 1. The other layers are very poor. To avoid training local optimality, pretraining and fine-tuning are adopted: training a network structure with only one hidden layer can learn the first-order features of the input residual data points.

Social network theory states that the actions of the subject depend to a large extent on the position of the community individuals in the structural system of social relations. The social network structure focuses on the analysis in terms of perceived centrality and other aspects, relying on the specific network association patterns to provide opportunities or restrictions to influence individual behavior. In virtual community social networks, members act as participating nodes with relatively fixed corresponding network sizes, so this paper chooses interactive relationships to achieve a replacement of network strength and network density, focusing on the fact that physical network technologies do not affect knowledge sharing.

Interaction relationship is mainly the frequency and depth of interaction among members in the virtual community social network, perceived centrality is the importance of members' status, and equivalence is the equal status of members in the virtual community social network. Based on Gravette's measurement scale, the scale was adjusted to include 4 items, 3 items for the perceived centrality dimension, and 2 items for the equivalence dimension.

The validity test is specifically a test of the validity of the questionnaire to determine whether the designed items are reasonable and whether they can effectively reflect the researcher's research objectives. Validity can be divided into three categories: content validity, criterion validity, and structural validity. Content validity is to justify the design of the questionnaire from various perspectives, and validation factor analysis also includes convergent validity and discriminant validity [15]. This layer of network structure after reaching the optimization starts training the next one. The content validity of this paper is very good after several minor modifications. The validity of the scale is generally reflected by the Person correlation coefficient, and the structural validity includes exploratory factor analysis and validation factor analysis. Factor analysis can be a good way to test the structural validity. First, to determine whether the scale is suitable for factor analysis, the KMO and Bartlett's spherical test are used, which can largely show the distribution of the data, and the KMO can largely show the degree of bias correlation among the variables.

The KMO values obtained after the test are close to 1, in which case Bartlett's spherical test is judged to be significant and the factor analysis is very appropriate, and the KMO values obtained after the test are less than 0.5 and the factor analysis is not appropriate. Applying SPSS22.0 to develop KMO values and Bartlett's spherical test, the KMO values were significantly greater than the minimum requirement of 0.5, including KMO values of 0.826 for self-efficacy, 0.870 for expected reward, 0.848 for social network structure, 0.847 for organizational trust, and 0.840 for knowledge sharing. Bartlett's spherical test was significant and factor analysis was very appropriate as shown in Table 1. Content validity is to demonstrate the rationality of questionnaire design from various perspectives, and confirmatory factor analysis also includes convergent validity and discriminant validity.

Among the above sales forecasting research methods, the traditional sales forecasting methods based on time series methods have a relatively simple calculation process, but the forecasting effect and the scope of application are relatively limited. Many scholars began to use machine learning methods to study sales forecasting, which improved the fitting ability of the forecasting model, and the combined forecasting model can further improve the forecasting effect. Many scholars have shown that there are factors that can be used for sales forecasting in addition to sales volume itself [16]. In addition to considering the change in historical sales, this topic also uses other marketing information affecting sales as independent variables for sales prediction, especially unstructured data such as pictures, which have been proven by many scholars' studies to be the most important marketing information online and have a huge impact on users' purchasing decision behavior.

## 4. Neural Networks for Cross-Border e-Commerce Sales Dynamic Prediction Model Design

BP neural network is essentially also a single-layer perceptron, which is also a multilayer feedforward neural network obtained by error backpropagation [17]. In this way, the influence of the early data in the sequence data on the later data can be further considered, so for the sequence data, the recurrent neural network often has a better effect. The core idea is to use the coordination mechanism of forwarding and backward propagation to continuously train the network and update the parameters. Forward propagation is like the normal flow of human blood circulation, inputting the neural network layer by layer to deeper neurons, and finally relying on the end neurons to output the results and do an error comparison with the actual output to judge whether there is an error and turn to the backward propagation stage of the error to update the parameter values when the results exist; reverse propagation is to propagate the output error back through the reverse network layer by layer to obtain the error information of each layer and use this as the basis for updating the weights of each layer.

The BP neural network uses the error function method to search for the optimal solution on the one hand and the random number method to assign the initial weights on the other hand, which makes the BP neural network have no global search capability and may easily fall into local optimum and slow convergence speed. Therefore, the particle swarm mechanism is used to improve the parameters in the BP neural network, and the optimal model performance is obtained by optimizing the training of the network model and continuously updating the speed and position of the particle swarm. Some defects of the basic particle swarm are mentioned in the previous section, and the improved particle swarm algorithm IPSO is proposed based on two improved operations of inertia weights and adaptive variation of genetic algorithm, which results in not only improving the global optimization seeking ability of the model and accelerating the convergence speed but also improving the prediction accuracy of the model.

In the past, most neural network information transfer is one-way and the input and output dimensions are fixed, which cannot solve some complex problems. Therefore, a new model structure of the recurrent neural network is introduced, which gives the neural network the function of memory and can obtain the influence of historical information on current prediction.

(1) $$\begin{array}{c} a_{l}^{t}=\overset{I}{\underset{i=1}{\sum }}w_{il}x_{i}^{t}-\overset{H}{\underset{h=1}{\sum }}w_{hl}b_{i}^{t}+\overset{C}{\underset{h=1}{\sum }}w_{hl}c_{i}^{t}\text{.} \end{array}$$

The processes of suitable data source selection, rigorous feasibility analysis, and fine data preprocessing greatly affect the final performance of the model. Therefore, to make the model accurately predict the sales of dishes, this part will introduce the three aspects of data sources, influencing factors processing, and data preprocessing.

The research object of this paper is the sales volume of the restaurant industry; however, the large differences in the dishes of stores in different business formats may seriously affect the generalizability of the model; at the same time, the daily sales volume of various dishes in most restaurant industries is proportional to the sales volume, and the sales volume of dishes can be further estimated from the daily dish sales; therefore, the sales volume of the restaurant industry is selected as the basic research object of this paper [18].

They jointly explained 48.9% of the variance of trust, and shared vision had the greatest impact; social interaction connection and shared vision had significant positive effects on reciprocity norm, and they jointly explained 43.6% of the variance of reciprocity norm. The direct relative returns of commodities and the relative returns of cross-selling have been mentioned earlier as two independent optimization objectives, and the direct relative returns of the rule corresponding to the combination of commodities are defined as the relative returns generated by the former set of commodities *Z*, i.e.,

(2) $$\begin{array}{c} Z_{1}=support\left(Z\right)\sum_{i\in X}p\left(i^{2}\right)\text{.} \end{array}$$

The cross-selling relative return corresponding to this rule is defined as

(3) $$\begin{array}{c} Z_{2}=\left(\frac{P\left(XY\right)}{P\left(X\right)}+\frac{P\left(\overset{-}{X}Y\right)}{\overset{-}{X}}\right)\overset{Y}{\underset{i=1}{\sum }}p\left(i^{2}\right)\text{.} \end{array}$$

A higher value of cross-selling relative return indicates that the correlation rule has a stronger effect on promoting the sales of the posterior item so that the candidate item combination not only has a high return of its own but also can promote the sales of other items.

Smoothing is performed on the nonstationary series. If the data series is not a random wandering trend, with a certain degree of regularity, the data is differenced, and the difference between the time series at a certain time interval is called delayed differencing. For the time series with periodic components, the delay may be periodic in period (width), the differencing process of the data is repeated, and the variance of the series is compared after all the several differencing, and the number of differencing with the smallest variance is selected as the result. At this time, part of the long-term trend and nonsmooth deterministic information of the original series is extracted, and all the time dependence of the original series will be eliminated [19]. The order of *p* and *q* is determined according to the AIC or BIC minimum criterion, and the estimates of the parameter coefficients are obtained by the least-squares estimation method. Least squares are a kind of estimation of parameters without first specifying the distribution of the series, with the principle of minimizing the sum of squares of the differences between the actual sales time series values and the model fitted values, with the help of an iterative method to make the residual sum of squares minimum as the goal. The value of a product drops rapidly over time, so even with strong promotions, sales of the product will still drop.

(4) $$\begin{array}{c} e_{t}=Y_{t}^{new}-\overset{\Gamma }{Y_{t}}. \end{array}$$

**Figure 2:**
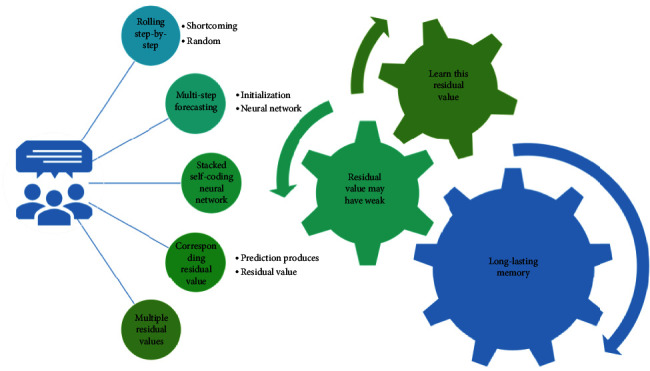
Sliding window calculation for input layer.

According to the literature, in most cases, rolling step-by-step forecasting is more effective than multistep forecasting, while one-step rolling forward forecasting is used to predict one value at a time to the corresponding residual value, and the correlation information between multiple residual values after accumulation cannot be fully explored. The stacked self-coding neural network can learn in real time and improve the shortcomings of random initialization of the neural network, when the prediction produces a residual value, it starts to learn this residual value, and the residual value may have weak and long-lasting memory (correlation) between the residual values, the corresponding accumulated residual sequence may be nonsmooth, and the neural network is adaptable to the nonsmooth environment and can fit the nonlinearity well. There is a strong adaptive nature, which effectively expresses the characteristics of nonlinearity and randomness among the residual series data, as shown in Figure 2.

The first term is designed as the mean square error among the residual data, the second term is designed as a sum of weights that decreases with time, decreasing the magnitude of the weights, and *a* is the weight decay term. The third term is the sparse penalty term, *β* is the sparse penalty term coefficient, and *p* is the average activation of the hidden layer node *j*, corresponding to the average activation of different subsequences. It is ensured that the distribution between the residuals learned by the computational model satisfies the approximately normal distribution, and the closer the distribution to the real white noise data, the better.

At this point, the neural network structure can make the linearly correlated or nonlinearly uncorrelated, relatively independent nodes in the hidden layer more sensitive to specific input features or residual data properties and explore the relevant linear region, which can be explored by learning the error of the objective function to obtain a neural network structure that can learn information.

Considering that the increase in the depth of the network structure can make the backpropagation fall into a local optimum, the deeper layers in the transfer of information lose some information, and the parameters of the layers near the output in the network are updated better, while the other layers are poor, to avoid training a local optimum. Training a network structure containing only one hidden layer that can learn the first-order features of the input residual data points, the network structure reaches optimization before starting to train the next one, and then these features are used as the input of the next network structure, using greedy learning ideas to complete the unsupervised pretraining of the overall stacked neural network structure.

**Figure 3:**
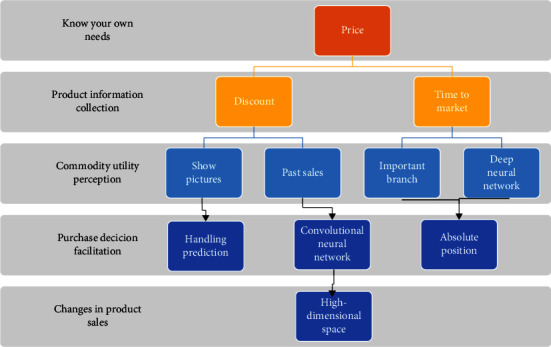
The formation stage of product sales and its influencing factors.

Deep learning has gradually developed into an important branch of machine learning. This may be since the values in this time are all higher on the first few days of the forecast date, resulting in higher values predicted by the model inertia. Compared with traditional machine learning methods, a deep neural network of deep learning can project data tasks to a high-dimensional space for processing, thus handling prediction tasks of higher complexity and fitting a sales curve that more closely matches reality [20]. In addition, since the convolutional neural network in deep learning is insensitive to the absolute position of objects in the picture data when processing them, deep learning can also effectively solve unstructured data of picture type, as shown in Figure 3.

Furthermore, for sequential data like sales, traditional time series methods and machine learning methods treat each moment of data as an independent feature, while recurrent neural networks in deep learning can record several previous inputs and states, thus being able to further consider the influence of earlier data on later data in sequential data, so recurrent neural networks tend to have better results for sequential data. Then use the corresponding tools and channels to transform the knowledge, and finally the knowledge acquirer internalizes and absorbs the knowledge, which itself is a process of knowledge learning.

When goods are exchanged with money, the price is usually involved in the transaction as the amount of money per unit of goods, and the price is usually regarded as the reflection of the value of the goods themselves. When users shop online, whether they have a target product in mind or not, their knowledge will make them know or expect the performance of the product they are browsing, so the price of the product will have a significant impact on deepening or evoking consumer demand [21]. Price is also a major factor that consumers consider during the product information-gathering phase, which in turn influences the subsequent product utility perception and evaluation phase.

## 5. Analysis of Results

### 5.1. Results of the Analysis of Factors Influencing Knowledge Sharing in Virtual Communities

The results of the goodness-of-fit test and the regression matrix of the regression model show that the tolerance, VIF, and Durbin-Watson are within reasonable limits and there is no multicollinearity among the independent variables. Conversely, when there is a crisis of trust within an organization, the quantity and quality of knowledge sharing can become seriously problematic. Based on the regression coefficients and significance levels, the independent variables all positively affect knowledge sharing, all four basic hypotheses are valid.

**Figure 4:**
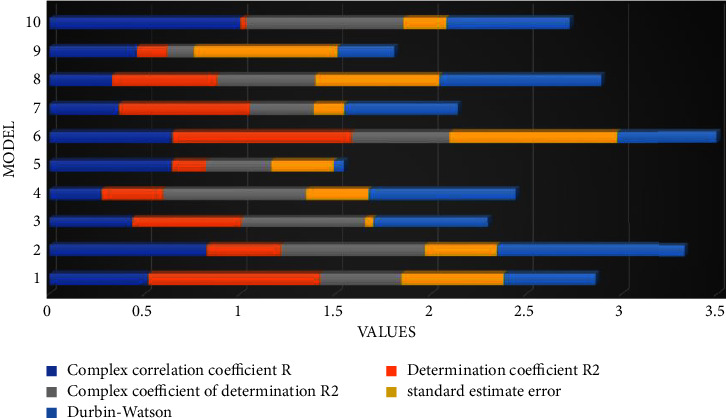
Goodness-of-fit test of the regression model of independent variables on explicit knowledge sharing.

This paper divides knowledge into explicit knowledge and tacit knowledge according to the characteristics of knowledge; i.e., the dependent variables are divided into two dimensions: explicit knowledge sharing behavior and tacit knowledge sharing behavior. To investigate whether there are differences in the effects of the respective variables under different knowledge characteristics, regression analysis is done separately in this paper.  Figure 4 shows the regression analysis of independent variables for explicit knowledge sharing. It can be found that self-efficacy and expected return do not enter the fitted model and have a positive impact on market orientation and inventory control. In recent years, the competition among e-commerce enterprises has become increasingly fierce. Therefore, it can be concluded that the positive effects of self-efficacy and expected returns on explicit knowledge sharing are not significant, while the effects of social network structure and organizational trust on explicit knowledge sharing are significant, and social network structure has a relatively greater degree of influence than organizational trust.

In this paper, the regression analysis was done separately for explicit and tacit knowledge sharing by extracting self-efficacy and expectation reward, and the results showed that whether it is explicit or tacit knowledge and whether it is self-efficacy or expectation reward of individuals in the knowledge sharing process, they all positively affect the knowledge sharing behavior, but the degree of explanation for explicit knowledge sharing alone is low. To occupy a favorable position in the market, it is necessary to accurately predict and judge the competitiveness of commodities.

**Figure 5:**
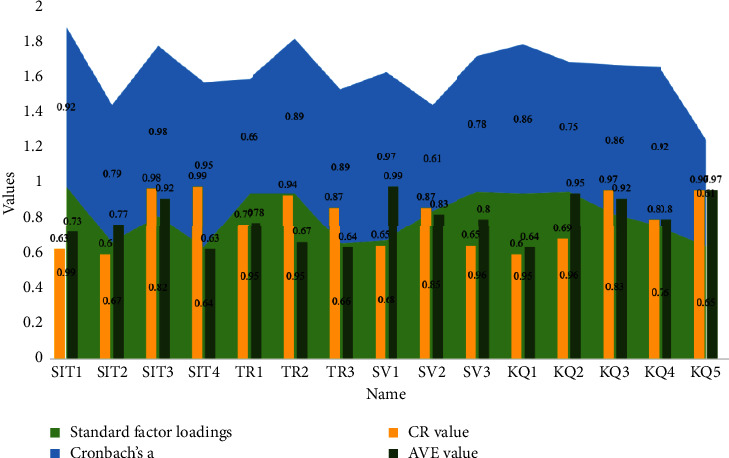
Standard factor loadings, Cronbach's alpha coefficient, CR values, and AVE values.

The reliability analysis of each research variable was conducted using SPSS, and Cronbach's *α* reliability coefficient was used to test the internal consistency of the questionnaire content. Validation factor analysis using AMOS showed that the fitted indicators (chi-squared degrees of freedom ratio = 2.061, GFI = 0.955, AGFI = 0.938, NFI = 0.954, IFI = 0.976, TLI = 0.970, CFI = 0.976, and RMSEA = 0.042) were all better than the standard values, indicating that the model was ideal. The standard factor loadings of each indicator were obtained to be above 0.7 and significant at the 0.001 level, with CR values greater than 0.7 and AVE values greater than 0.5, indicating that the scale has good convergent validity. The more rigorous AVE method was used to test the discriminant validity of the scale, and the results showed that the square root of the AVE values of each variable was greater than the corresponding correlation coefficients, indicating that the scale has good discriminant validity, as shown in Figure 5.

Social interaction bonding and shared vision have significant positive effects on trust and together explain 48.9% of the variance of trust, with shared vision having the largest effect; social interaction bonding and shared vision have significant positive effects on reciprocity norms and together explain 43.6% of the variance of reciprocity norms, with social interaction bonding having the largest effect; social interaction bonding has significant positive effects on a shared vision, and together they explain 27.9% of the variance of a shared vision. Social interaction connection has a significant positive effect on shared vision and jointly explains 27.6% of the variance of shared vision; trust, reciprocity norms, and shared vision all have a significant positive effect on knowledge quality and jointly explain 54.9% of the variance of knowledge quality, with the effect of reciprocity norms being the largest and the effect of social interaction connection on knowledge quality not being significant.

Based on this research objective, the relationship between social interaction linkages, shared vision and trust, and reciprocity norms, the relationship between social interaction linkages and shared vision, the relationship between social interaction linkages, trust, reciprocity norms, shared vision, and knowledge quality, and the mediating role of trust, reciprocity norms, and shared vision in academic virtual communities were explored with due consideration of the characteristics of academic virtual communities. A reasonable prediction will bring huge economic benefits and maintain the stable development of the market. After identifying the research questions, a questionnaire was designed using the question items that have been used in the existing domestic and international literature, combined with the results of a small-scale pretest. Based on this, an empirical study was conducted with questionnaires from users who had participated in academic virtual communities. SPSS and AMOS statistical analysis software were used to analyze the data of the collected valid samples, including descriptive statistics, reliability analysis, hypothesis testing of the model, and mediating effect testing.

### 5.2. Analysis of Dynamic Prediction Results of Cross-Border e-Commerce Sales by Neural Networks

Most machines learning training is based on the batch gradient descent method. The batch gradient descent method will input all the training data into the model for each training, and for the overall data, there may be saddle points, and the training of the model may fall into the saddle points and stop training, which is the local optimum problem. An effective method is to randomly select a batch of data from the data for each training. Based on this training method, although it is almost impossible for the training process to directly descend in the direction of a large gradient, it makes it more likely to jump out of the saddle point, i.e., the local optimal point, during training. Moreover, for neural networks, the use of random batch gradient descent can reduce the training overhead due to its large number of weights, many computational steps, and the time required to bring in all the data for each optimization.

To avoid overfitting the training data, the data set is first divided into training and test data, and the loss values of both training and test data are observed. It shows the characteristics of short sales life cycle and easy depreciation. When the loss value of the test data no longer decreases, training is stopped. However, since the optimization process of stochastic batch gradient descent does not always optimize along the overall optimal direction, it is necessary to record the optimal loss value of the test data and stop training when *n* training sessions do not result in a better loss value and the loss value of the training data keeps decreasing.

**Figure 6:**
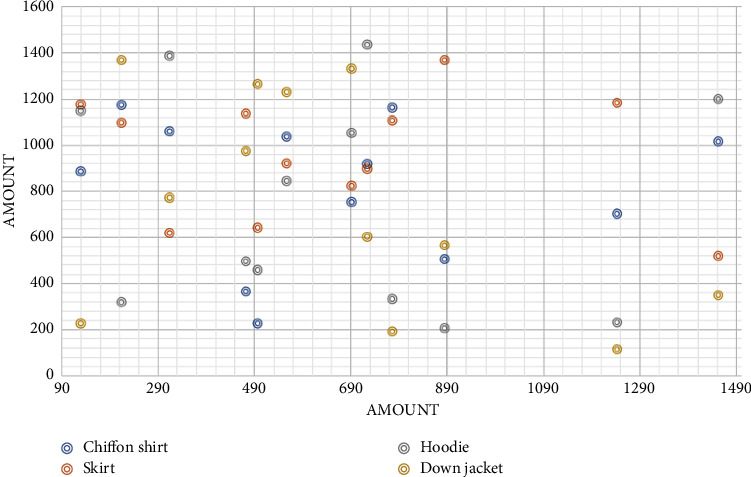
Sales trends of different products.

In this paper, according to the different types of products of this enterprise, the sales of its products are counted by month, and four products with similar annual sales among the main products it sells are selected to study their sales changes and make a sales change trend graph, as shown in Figure 6. Different types of products showed different sales trends. The sales of down jackets, as cold-weather clothing, gradually increased after September when the weather became colder.

We can see that small promotion can lead to an increase in sales, but as the discounts increase, the sales decrease. The reason is that although price reduction will lower the economic cost of users and motivate them to make purchases, too much price reduction will also reduce the perceived quality of the product and increase the perceived risk of users, thus preventing them from making purchase decisions. In addition, large promotions are mostly seasonal clearance for companies, when price cuts are made to address the buildup of inventory caused by inaccurate sales forecasts in the past period. Apparel products are short life cycle products, and the value of products declines rapidly with time, so even with strong promotional actions, product sales will still decline. However, the trend and scatter plots show that the correlation between product sales and discounts is not that significant, so the relationship between product discounts and sales needs further consideration of other factors.

After the initialization of the neural network, we plot the distribution of the output of all hidden layers to prevent the Dead ReLu phenomenon. For some fast fashion products, such as shoes and hats, apparel, and fashion, it is more challenging to forecast the demand because the product life cycle is relatively short. According to the distribution of the network output, we can see that the initialization of this paper is effective, and many neuron nodes are in the active state. Taking the distribution of three convolutional layers of image data processing as an example, some neurons in the first convolutional layer are activated while some neurons are inhibited, which shows that the first convolutional layer maintains the nonlinearity of the neural network output, and the later higher convolutional layers do not have many zero outputs.

**Figure 7:**
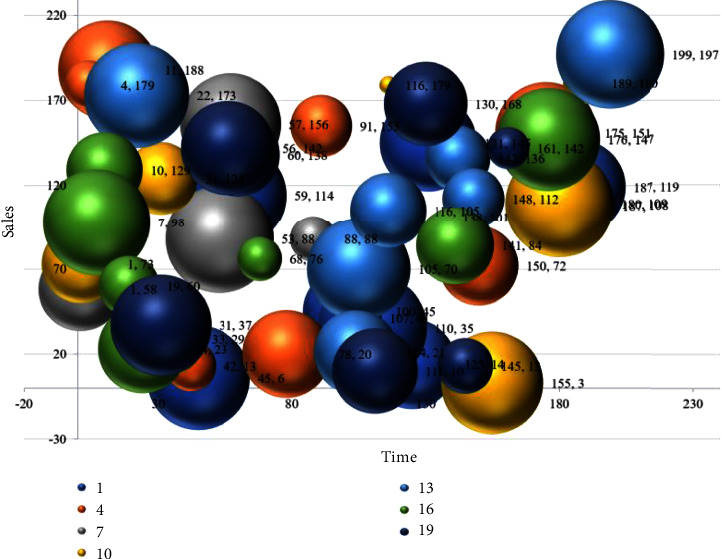
Price prediction results.

A reasonable initialization method can help avoid the gradient disappearance problem of neural networks with ReLu as the activation function but cannot avoid the gradient explosion problem caused by more layers of the neural network. In this paper, we use the gradient cropping method to avoid the problem of gradient explosion, which makes the weights of the deep neural network repeatedly wobble and fail to converge. Gradient clipping is to limit the gradient deinterval after the gradient calculation of each layer to avoid excessive gradients. After several experiments, it is found that setting the maximum and minimum values of the gradient to [−0.01, 0.01] can avoid the gradient explosion while keeping the training speed as much as possible, as shown in Figure 7.

From the overall situation, there are two trends in the performance effect of the model. The first one is that the model using particle swarm optimization performs better than the single model in each index, and in turn, the model using improved particle swarm optimization performs better than the base particle swarm optimization model in each index; the second one is that the model of LSTM series outperforms the model of BP series in general. In terms of specific models, the IPSO-LSTM algorithm has the best results, corresponding to the smallest MAPE, RMSE, and MSE metrics and the largest 2R goodness-of-fit metric, which also largely indicates that our improved particle swarm approach is effective and can improve the prediction accuracy.

All six models can fit the true value trend, but the predicted values are higher than the actual values during the peak hours of 6:00 p.m. to 7:00 p.m. This may be because the values are all higher during this period in the days before the prediction date, resulting in higher values predicted by the model inertia. In terms of the individual models, the IPSO-LSTM algorithm model is better able to fit the trend of the true values and can accurately portray the predicted changes in sales, while the single model BP neural network is the worst fit and deviates far from the actual values.

## 6. Conclusion

Under nontransactional virtual community codependency, users continuously participate to maintain dependency, increase loyalty to the virtual community, and continue knowledge sharing. To maintain identifiable identity, public self-awareness, group norms, and community participation to gain recognition and affirmation, users will invest more energy and thus enhance their loyalty to virtual communities; similarly, they will be more willing to sustain knowledge sharing in virtual communities. Forecast the demand and sales of fast fashion products. The biggest product features of fast fashion products are quick feedback on show design, shorter product life cycle, and higher frequency of updating product categories and types. In contrast, the higher the loyalty of nontransactional virtual community users, the more they want the virtual community to operate more successfully and thus continue to share knowledge in the virtual community. Given the importance of product reorder rate, a Poisson regression model is used to calculate the reorder rate of different products in the case and the factors that play a strong influence on the reorder rate according to the formula, which lays the data foundation for subsequent product combination recommendation and product sales prediction. A nested neural network with BP, LSTM, and Verhulst models based on the AP algorithm is constructed. The BP neural network combines the current independent variables to predict sales, the LSTM neural network explores the influence of historical data on sales, and the Verhulst model can predict sales based on the growth trend of the dependent variable. The AP algorithm is used to forecast and analyze the sales of goods, and the error comparison proves that nested neural networks are more accurate in predicting the sales of goods.

## Figures and Tables

**Table 1:** KMO and Bartlett's spherical test of variables.

Name	KMO	Bartlett's sphericity test		
		Approximate chi-square	df	Sig.
Self-efficacy	0.192	0.237	0.117	0.255
Expected return	0.221	0.35	0.222	0.304
Organizational trust	0.367	0.277	0.327	0.168
Social network structure	0.27	0.23	0.194	0.389
Knowledge sharing	0.264	0.377	0.112	0.142
General table	0.208	0.292	0.317	0.361

## Data Availability

The data used to support the findings of this study are available from the corresponding author upon request.
